# Editorial: Case reports in nephrology

**DOI:** 10.3389/fmed.2023.1278138

**Published:** 2024-01-29

**Authors:** Sree Bhushan Raju, Manish Rathi, Sandeep Mahajan

**Affiliations:** ^1^Department of Nephrology, Nizam's Institute of Medical Sciences, Hyderabad, India; ^2^Department of Nephrology, Post Graduate Institute of Medical Education and Research (PGIMER), Chandigarh, Haryana, India; ^3^Department of Nephrology, All India Institute of Medical Sciences, New Delhi, India

**Keywords:** CKD, AKI, nephrotic syndrome, genetic kidney diseases, pregnancy, breast milk, dialysis, COVID vaccine

Case reports are an important source of information and learning in the medical literature. These are also usually the first publications by many doctors in their academic careers. Though, in the era of evidence-based medicine, case reports are lower down in the hierarchy of evidence, they form a unique way of sharing experiences and anecdotal associations that can then be subjected to more rigorous analysis. In this issue, we have compiled unique and interesting case reports related to the field of nephrology that will be relevant in clinical practice and will be of interest to nephrologists and physicians alike.

## Pregnancy and renal diseases

There has been a steady increase in the success rate of pregnancy among patients on dialysis since the 1980s (1). Improvements in dialysis technology and adequacy along with better drug therapy and nutritional management have made this possible. Pregnancy just before the initiation of dialysis and in the 1^st^ year of it is likely to be successful.

In an interesting case report, Mambap et al. reported a successful pregnancy while on dialysis for 11 years. The pregnancy was maintained until 36 weeks, when a healthy 2,270 g female baby was delivered by elective cesarean section for tight nuchal cords and intrauterine growth retardation. The mother maintained well in the last follow-up of 4 years, and the child was healthy.

In another case report by Kondakova et al., breast milk was evaluated before and after dialysis in a 31-year-old woman who had a successful delivery while on dialysis. The authors did not reveal an optimal time interval for breastfeeding a baby. Furthermore, they opined that breastfeeding is not advisable since the concentration of nutrients is low, and the content of toxic substances exceeds the permissible limits.

One of the major renal emergencies during pregnancy is renal colic due to calculous renal disease. Cystinuria is a rare genetic disorder that is characterized by excessive urinary excretion of cystine. It predominantly affects men compared to women. Ivandic et al. described a case of a 38-year-old woman with cystinuria who manifested cystine stones during her third pregnancy, with a very complicated course and several urological interventions during pregnancy due to the formation of new stones and worsening of kidney function. Despite all the complications during the pregnancy, she successfully delivered a healthy girl.

## Genetic diseases and the kidney

Autosomal dominant tubulointerstitial kidney disease due to UMOD mutations (ADTKD-UMOD) is a rare condition associated with end-stage kidney disease (ESKD). It is majorly seen among adult men. Li et al. reported a 13-year-old young girl with unexplained chronic kidney disease and no positive family history. Trio whole-exome sequencing confirmed that she carried a *de novo* heterozygous mutation c.280T>C (p.Cys94Arg) in the UMOD gene. Due to a lack of targeted therapy, she was treated with conservative therapy of chronic kidney disease (CKD). This case illustrates the value of whole-exome sequencing in a patient in whom the cause of CKD is not clear, especially during childhood.

## Infection and the kidney

The most common bacterial infection following renal transplantation is urinary tract infection (UTI), including pyelonephritis, ranging from 7 to 86% of all renal transplant recipients (2). It is associated with excess risk of graft loss and death. Emphysematous pyelonephritis (EPN) is a severe, acute necrotizing infection that is defined by the presence of gas in the kidney parenchyma. Multiple case reports have described both the radiologic features and clinical course of native kidney EPN. Abu Jawdeh et al. reported a case of EPN in a renal transplant recipient. It was further complicated by multiorgan failure and finally required graft nephrectomy. This case highlights the poor outcome of EPN in the post-renal transplant setting. Transplant kidneys, being the solitary functioning kidney, may not respond well to antibiotic therapy when they develop EPN and may require graft nephrectomy, resulting in loss of the functioning graft. This case emphasizes the need for definite strategies aimed at reducing UTI and pyelonephritis in renal transplant recipients, which will further improve transplant outcomes in the long run.

## AKI

Globally, 13 million people worldwide are thought to be affected by acute kidney injury (AKI) every year. It is well established that AKI is associated with adverse outcomes, including development or worsening of CKD, cardiovascular events, and mortality. Several complications have been reported in association with AKI. Community-acquired AKI has a varied etiology and milder course compared to hospital-acquired AKI (3). Indigenous substances are one of the frequent causes of AKI, especially in the developing world, where there is high dependence on alternative medicines and faith healers. Torabi Jahromi et al. reported a rare case of a 35-year-old woman presenting with renal arcuate vein thrombosis (RAVT) and acute kidney injury (AKI) following upper respiratory tract symptoms and toxic substance ingestion. Renal biopsy suggested venous thrombosis in the renal arcuate veins. The patient's symptoms resolved following anticoagulation with apixaban, a direct oral anticoagulant. Though there are a limited number of studies showing the concurrent presentation of RAVT and overt AKI in patients following ingestion of nephrotoxic agents, this case illustrates the necessity of the early evaluation of etiology and appropriate management to prevent progression to CKD.

Wang L. et al. reported AKI following a medicinal herb leading to acute oxalate nephropathy (AON). This case highlights the need for a thorough medication history, including the history of use of medicinal herbs in all patients with community-acquired AKI. The use of medicinal herbs with unknown oxalate contents increases the risk of AON and should be avoided.

## Complications associated with CKD

Tripterygium wilfordii is a traditional Chinese herbal medicine that is used to treat several diseases, including CKD, rheumatic autoimmune disorder, and skin disorders. Zhang et al. reported the first case of a 50-year-old Chinese female with ESRD who developed severe bone marrow suppression after taking a short-term normal dose of a T. wilfordii-containing decoction. She died of sepsis and septic shock, although timely therapeutic measures (e.g., stimulating hematopoiesis, anti-infection treatment, and hemodialysis) were administered. This case contradicts the notion that side effects of Chinese herbs on the hematopoietic system are non-lethal and points out that patients with ESRD are at higher risk of such complications.

CKD leads to defects in divalent ion metabolism, leading to secondary hyperparathyroidism characterized by hypocalcemia and hyperphosphatemia. Sevelamer carbonate is the most widely used non-calcium-based phosphate binder. Gastrointestinal (GI) injury associated with sevelamer use is a documented adverse effect but is underrecognized as a cause of life-threatening GI complications. Fistrek Prlic et al. reported the case of a 74-year-old woman taking low-dose sevelamer with serious GI adverse effects causing colon rupture and severe GI bleeding. This case documents an important adverse effect of a frequently used drug in nephrology practice and cautions against its use in elderly people with risk factors for GI complications.

## Nephrotic syndrome

Minimal change disease (MCD) is one of the common causes of idiopathic nephrotic syndrome (INS), accounting for 10–20% of INS in adults. Rituximab has been used successfully in patients with autoimmune hemolytic anemia (4). Zhuang et al. reported the case of an adult patient with refractory MCD complicated with β-thalassemia minor and accompanied by autoimmune hemolytic anemia. The patient had a frequently relapsing course with steroids and ultimately achieved clinical complete remission after the administration of rituximab. Moreover, anemia due to mild β-thalassemia also recovered to normal. The disease situation remained stable during the 36 months of follow-up. These findings suggest that rituximab may contribute to the improvement of steroid-dependent or frequently relapsing MCD and anemia in β-thalassemia minor accompanied by autoimmune hemolytic anemia.

Idiopathic multicentric Castleman disease (iMCD) is a systemic and polyclonal lymphoproliferative disease, leading to the overproduction of interleukin-6 (IL-6), that involves multiple organs, including the kidneys. Previous reports suggested that excessive IL-6 actions in iMCD have a causal relationship with the development of diverse histopathological renal manifestations, which cause nephrotic syndrome. Kojima et al. reported a series of three cases of nephrotic syndrome due to iMCD that help to delineate the importance of early and continuous therapy with the anti-interleukin-6 receptor antibody tocilizumab. All three patients presented with nephrotic syndrome, and renal biopsy showed diffuse mesangial and endocapillary hypercellularity without immune deposits, along with AKI in one and immune-complex glomerulonephritis with AKI in another. The third case was diagnosed with nephrotic syndrome secondary to membranous glomerulonephritis, with IgE antibodies to tocilizumab, and was therefore treated with prednisolone alone. In contrast to the first two cases, the third progressed to ESRD. This case series suggests the necessity of maintaining clinical vigilance for iMCD as a possible underlying component of nephrotic syndrome and the prompt initiation and continuous administration of tocilizumab.

Nephrotic syndrome with dual etiology in a single patient is not a usual entity. Wang Y. et al. described a 39-year-old male patient with IMN combined with immunoglobulin light-chain amyloidosis nephropathy who presented with nephrotic syndrome. Renal pathology revealed MN. A positive Congo red staining and the pathognomonic apple-green birefringence under cross-polarized light were considered to be associated with amyloid nephropathy. Immunofluorescence showed that the λ light chain was positive. His serum was negative for antibodies against the Phospholipase A2 receptor (PLA2R), but PLA2R was present in the renal tissue. It is very interesting to note the dual pathology and management becomes crucial in such situations.

Association between nephrotic syndrome and malignancies is not unusual, but more cases have been reported with membranous nephropathy. Cai et al. reported a patient with MCD simultaneously associated with papillary thyroid carcinoma (PTC). After surgery, MCD remitted rapidly and completely, strongly suggesting the diagnosis of MCD secondary to PTC. This case highlights the importance of tumor screening wherever indicated and avoiding conventional therapy with steroids in cases of MCD.

## COVID vaccine and the kidney

There are numerous reports of renal diseases occurring after COVID vaccination (5). Zamoner et al. reported a case of anti-neutrophilic cytoplasmic antibody (ANCA)-positive crescentic glomerulonephritis 5 days following vaccination with the Oxford–AstraZeneca COVID-19 vaccine in a 58-year-old female patient. She was treated with steroids and cyclophosphamide, leading to the stabilization of the creatinine. Early detection and prompt institution of therapy are key to restoring complications following COVID-19 vaccination.

## Author contributions

SR: Conceptualization, Data curation, Formal analysis, Investigation, Methodology, Project administration, Resources, Software, Supervision, Validation, Visualization, Writing – original draft, Writing – review & editing. MR: Supervision, Writing – original draft. SM: Conceptualization, Supervision, Writing – original draft.

## References

[1] Manisco G Poti M Maggiulli G Di Tullio M Losappio V Vernaglione L. Pregnancy in end stage renal disease patients on dialysis: how to achieve a successful deliver. Clin Kidney J. (2015) 8:293–9. 10.1093/ckj/sfv016 26034591 PMC4440463

[2] Graversen ME Dalgaard LS Jensen-Fangel S Jespersen B Østergaard L Søgaard OS. Risk and outcome of pyelonephritis among renal transplant recipients. BMC Infect Dis. (2016) 16:264. 10.1186/s12879-016-1608-x 27287058 PMC4901406

[3] Sawhney S Fraser SD. Epidemiology of AKI: utilizing large databases to determine the burden of AKI. Adv Chronic Kidney Dis. (2017) 24:194–204. 10.1053/j.ackd.2017.05.001 28778358 PMC5648688

[4] Barcellini W Fattizzo B. How I treat warm autoimmune hemolytic anemia. Blood. (2021) 137:1283–94. 10.1182/blood.2019003808 33512406

[5] Fenoglio R Lalloni S Marchisio M Oddone V De Simone E Del Vecchio G. New onset biopsy-proven nephropathies after COVID vaccination. Am J Nephrol. (2022) 53:325–30. 10.1159/000523962 35354140 PMC9059008

